# Emotion Socialization Under One Roof: How Parental Response Patterns Shape Adolescent Emotional Well-Being

**DOI:** 10.3390/bs15080999

**Published:** 2025-07-22

**Authors:** Huiyuan Gao, Yue Guan, Wenyue Pei, Yuhan Gao, Jiayue Mao, Suqun Liao, Can Zeng

**Affiliations:** 1 School of Psychology and Cognitive Science, East China Normal University, Shanghai 200062, China; 10220330415@stu.ecnu.edu.cn (W.P.); gyh1281615610@163.com (Y.G.); 2 School of Nursing, Hubei University of Chinese Medicine, Wuhan 430065, China; guanyueeic@163.com; 3 Department of Applied Psychology, New York University, New York, NY 10012, USA; jm7724@nyu.edu; 4 Department of Psychology, Shaoguan University, Shaoguan 512158, China; liaosq@sgu.edu.cn (S.L.); zengcan@sgu.edu.cn (C.Z.)

**Keywords:** latent profile analysis, emotion socialization, adolescents, depressive symptoms, regulation

## Abstract

(1) Background: This study used latent profile analysis (LPA) to investigate family patterns of paternal and maternal responses to adolescents’ discrete emotions (happiness, sadness, and anger) and examined the relationship between these profiles and demographic factors, as well as adolescents’ emotion adjustment (emotion regulation and depressive symptoms). (2) Methods: A sample of 666 adolescents reported parental responses and their emotional adjustment; their mothers provided family information. (3) Results: (a) The LPA identified four profiles for adolescent happiness, including high enhancing but low dampening and neglect from both parents (Consistent Supportive); low enhancing but high dampening and neglect from both parents (Consistent Unsupportive); low to moderate scores on each response from both parents (Consistent Disengaging); and high maternal dampening and neglect but relatively low scores on the paternal response (Inconsistent). There were two profiles for sadness (Consistent Supportive, Consistent Unsupportive) and three for anger (Consistent Supportive, Consistent Unsupportive, Consistent Disengaging). (b) Parents with boys, higher incomes, better education, and greater marital satisfaction were likely to be classified into the Consistent Supportive profile across emotions. (c) When adolescents perceived their parents with the Consistent Supportive profile, they would show the best emotional adjustment; while for parents with the Inconsistent profile (for happiness) and the Consistent Unsupportive profile, the adolescents had the poorest outcome. Interestingly, adolescents who perceived their parents as fitting the Consistent Disengaging profile (especially for anger) exhibited comparatively less adverse adjustment. (4) Implications: A person-centered approach highlights different patterns of emotion socialization, underscores the importance of fostering parental cooperation and supportive responses to adolescents’ happiness, and suggests that joint disengagement from anger may promote healthier emotional development.

## 1. Introduction

Parental emotion socialization (ES) influences how children experience, express, and regulate emotions, thus contributing to child socio-emotional development (Eisenberg et al., 1998a; Eisenberg, 2020; Morris et al., 2007). Adolescence is a particularly critical period for emotional development, marked by heightened vulnerability to emotional problems such as depression and emotion dysregulation. Consequently, understanding how parents’ responses to adolescents’ emotions shape their psychological adjustment has become a significant area of inquiry. Existing research on ES has predominantly focused on parental reactions to negative emotions (e.g., sadness, anger) while overlooking their reactions to positive emotions (e.g., happiness; Fredrickson, 1998, 2013). Furthermore, most studies have centered on maternal socialization practices, neglecting the unique and complementary roles of fathers (Cassano et al., 2007; McElwain et al., 2007). This limited scope leaves a significant gap in understanding how parents jointly influence adolescents’ emotional outcomes. To address these gaps, this study conducted latent profile analysis (LPA) to identify patterns of parental reactions to discrete emotions in two-parent households and explored the key correlations informed by previous research, including demographics (i.e., adolescents’ gender, mothers’ education, marital satisfaction, and family income) and outcomes (i.e., emotion regulation and depressive symptoms).

### 1.1. Parental Emotion Socialization

Parental emotion socialization refers to the parenting practices that reflect parental beliefs and goals concerning their children’s emotional experience, expression, and regulation. Such practices encompass three key components: parents’ reactions to children’s emotions, discussions of emotions, and parental expression of emotions (Eisenberg et al., 1998b). Parents may react to children’s negative emotions in various ways, which can be deemed as supportive or unsupportive. Supportive reactions, such as emotion coaching, convey parents’ accepting and encouraging attitudes toward children’s emotional displays. In addition to emotion coaching, supportive parental reactions may be presented through more specific and diverse pathways, such as praising, offering comfort (i.e., reward), and engaging in joint problem-solving. It has been documented that those supportive reactions are associated with fewer children internalizing problems (Klimes-Dougan et al., 2007; O’Neal & Magai, 2005), greater positive emotion experience, and better emotion expression ability (Garside, 2004). In contrast, parents’ unsupportive practices, such as dismissing or neglecting reactions, can exacerbate emotional dysregulation and psychological distress (Garside & Klimes-Dougan, 2002; Lunkenheimer et al., 2007). Nevertheless, previous studies on parental reactions to children’s emotions were predominantly conducted with samples from Western countries, with a limited number of studies evaluating it in other cultural groups (i.e., China). 

Individuals from collectivistic cultures, driven by values emphasizing social harmony, often employ emotion restraint strategies more frequently than those from individualistic cultures (Matsumoto et al., 2008). In China, the Analects proverb “To restrain oneself and return to propriety” (ke ji fu li; Analects 12-1) encapsulates Confucian ideals of self-control and harmony, which underpin ES practices aimed at relational cohesion (Ding et al., 2024; Luo et al., 2013). Among these culturally influenced practices, two parental reactions have particularly attracted attention and debate: temporarily withholding communication (especially regarding children’s anger; Raval & Martini, 2011) and minimizing the seriousness of emotional situations (Shi et al., 2024a; Tao et al., 2010). Although Chinese parents often display these unsupportive reactions more frequently than Western parents (Yang et al., 2020), emerging evidence suggests they are not necessarily detrimental to children in Eastern contexts. For instance, parental minimization showed no negative link to Chinese children’s adjustment (Tao et al., 2010), even correlating positively with children’s functioning (Shi et al., 2024a). Similarly, temporary withholding of communication (i.e., not talking to the child for a brief period) occurs more frequently in well-adjusted rather than maladjusted families (Raval & Martini, 2011). Scholars suggest that Chinese children, influenced by Confucian filial piety, may interpret these parental strategies positively—as expressions of parental care that provide opportunities for self-reflection, emotional recovery. These strategies might be particularly valuable for early adolescents due to their rapidly developing cognitive and reasoning abilities. Thus, recognizing these cultural nuances is essential for understanding how parental reactions influence adolescents’ adjustment. We therefore surveyed Chinese seventh graders to examine associations between culturally embedded parental responses to adolescents’ emotions and to explore whether such reactions would show weaker negative associations with adjustment within this cultural context.

### 1.2. Socialization of the Different Emotions

From a functionalist perspective, each emotion serves a distinct social function (Barett & Campos, 1987), and parents tend to tailor their responses accordingly (O’Neal & Magai, 2005). However, most studies have conceptualized negative emotions broadly, overlooking distinctions among discrete emotions. Anger typically arises when obstacles impede goal attainment and receives fewer supportive responses; whereas sadness, as a nonhostile emotion, follows perceived loss and evokes empathy and nurturing from parents (Tomkins, 2008). Empirical studies demonstrate that parents provide more empathy, comfort, and practical support for sadness than for anger (O’Neal & Magai, 2005; Shipman & Zeman, 2001; Zeman & Shipman, 1997). This pattern is especially pronounced in collectivist cultures that prioritize harmony; for example, Indian mothers regard expressions of anger as less acceptable than sadness (Raval et al., 2016; Raval & Martini, 2009). Thus, this study examines Chinese adolescents’ negative discrete emotions, hypothesizing that parental responses will be less supportive toward anger compared to sadness. 

Increasingly, scholars recognize how crucial the socialization of positive emotion is for development. Parents’ enhancing/supportive behaviors generally relate to better emotion regulation and fewer depressive symptoms (Katz et al., 2014; Shi et al., 2024a; Moran et al., 2018; Yap et al., 2008; Yi et al., 2016), whereas dampening/unsupportive responses do not, particularly among Chinese adolescents (Shi et al., 2024a; Song et al., 2019). Although most work focused on the general positive emotions, Russell’s (1980) circumplex model delineates emotions by valence (pleasantness) and arousal (activation). Happiness (high pleasure, high arousal) and anger (low pleasure, high arousal) both share heightened arousal and potential for interpersonal contagion, whereas sadness (low pleasure, low arousal) differs substantially. 

Building on this framework, we speculate that patterns of parental response to happiness and anger may be more similar due to their shared high arousal, whereas responses to happiness may differ more markedly from those toward sadness. Nonetheless, happiness remains distinct because it is often viewed as a desirable internal state and elicits emotion upregulation strategies that broaden children’s thought–action repertoires as described by broaden-and-build theory (Fredrickson, 1998). Laboratory studies further illustrate that parents respond to children’s happiness with greater tonal warmth, emotional coaching, and flexibility (i.e., employing a broader range of strategies) than they do to anger or distress (Suveg et al., 2008; Lunkenheimer et al., 2007, 2012). Thus, the current study examines ES by including happiness alongside sadness and anger, and we expect that parental reactions to happiness will be notably diverse and spontaneous, as they may not require the heightened vigilance typically associated with negative emotions.

### 1.3. Person-Centered Approach to Parental Emotion Socialization in Families

Traditionally, ES research has focused on mothers as primary caregivers, but recent studies highlight the important yet distinct role of fathers. Fathers tend to respond to children’s negative emotions with more minimizing, neglecting, or punishing behaviors, while mothers are generally more constructive (Cassano et al., 2007; Eisenberg et al., 1996). In Chinese cultural contexts, fathers are often seen as strict and emotionally reserved (Li, 2021); Wang et al. (2019) likewise found that paternal disengagement predominates in China, whereas a cross-cultural study reported particularly high levels of unsupportive responses among Chinese mothers (Song et al., 2019). Despite lower paternal involvement in children’s emotional lives, fathers’ reactions significantly influence children’s emotional arousal (Van Lissa et al., 2019), especially among depressed adolescents (Allen et al., 2012). Parental responses vary by emotion type: Hooven et al. (1995) observed that fathers attend more to children’s anger, while mothers were more concerned with sadness. Unsupportive reactions in these respective domains may thus exert stronger effects. Consistent with this, Sanders et al. (2015) found that paternal unsupportive responses to anger, rather than sadness, predicted higher levels of children’s depressive symptoms, whereas the reverse was true for mothers. These findings underscore the importance of considering both parent gender and emotion type in the process of ES. 

However, from a family systems perspective, mothers and fathers jointly form a subsystem where their combined emotional responses influence child outcomes. Yu et al. (2015) explored this dynamic by creating an interaction term and found that Chinese children benefited most when both parents responded supportively; conversely, divergent parental reactions (a highly supportive father paired with a less supportive mother) were linked to more internalizing problems. Although this study employed a variable-centered approach, it highlighted the critical importance of parental consistency in emotion socialization, expanding prior research by examining how joint parental patterns influence adolescent development. 

Recently, researchers have increasingly adopted person-centered approaches (i.e., cluster analysis) to better capture family heterogeneity by identifying distinct patterns of parental responses. Miller-Slough et al. (2018) identified three ES classes among U.S. two-parent families: two classes reflecting parental consistency—“supportive” (high support from both parents) and “unsupportive” (high nonsupport from both parents)—and one labeled “father dominant,” reflecting divergences between parents, with high paternal but low maternal involvement. Similarly, Zhu and Dunsmore (2023) classified Chinese families into five patterns combining parental ES and family functioning, highlighting consistent (both supportive or unsupportive) and discrepant classes (e.g., engaged father/mother). Both studies found that children with consistently supportive parents showed better adjustment, those with unsupportive parents fared worst, and the discrepancy pattern had intermediate outcomes. Childrearing agreement and parental encouragement foster a stable emotional environment for secure attachment (Feinberg, 2003). Therefore, we anticipated that Chinese parents would also exhibit these consistent and discrepant ES patterns, with consistently supportive responses being particularly beneficial for child adjustment outcomes.

Moreover, we observed a “balance/diffuse” pattern first identified by Zhu and Dunsmore (2023) in Chinese two-parent households and also detected by Wang et al. (2019) in a Chinese father sample (where it was labeled “disengaged” via LPA). This profile is characterized by uniformly low levels of emotional responsiveness—reflecting relative passivity on both parents’ part, or especially the father’s—and appears common in Chinese families. Interestingly, both studies found no differences in children’s emotional adjustment between the disengaging and supportive profiles. Some researchers have attributed this to cultural norms of emotional restraint, whereby less-harsh unsupportive reactions (e.g., withholding communication or minimization) are not linked to poorer child outcomes, as mentioned before. Accordingly, we expected that this disengaged emotion socialization pattern would emerge in our sample and that adolescents exposed to it would demonstrate levels of emotion regulation and depressive symptoms comparable to those of peers with supportive parents.

Although person-centered studies (i.e., cluster analysis, LPA) have advanced our understanding of ES, they focus on broad negative emotions and rarely address positive ones like happiness. Moreover, predictors of parental ES patterns, including parents’ (i.e., marital quality and socioeconomic status) and children’s characteristics (i.e., gender), remain inadequately explored. Specifically, King et al. (2023) linked interparental conflict with a dismissive parenting profile, but marital satisfaction has been overlooked. Socioeconomic status shows mixed results: while higher family income is linked to parents’ active engagement style (“hyper-engaged”; Sosa-Hernandez et al., 2020), other studies did not find any significant relationships (King et al., 2023). Few studies have included child gender as a factor, though some empirical studies using traditional analyses suggest that parents encourage sadness in girls and tolerate more anger in boys (Chaplin et al., 2005; Garside & Klimes-Dougan, 2002). This study thus used adolescent-reported ES and explored the role of parental characteristics and child gender in ES patterns.

### 1.4. The Current Study

The present study drew on adolescents’ perceptions and used LPA to explore (1) family patterns of Chinese fathers’ and mothers’ socialization practices regarding adolescents’ happiness, sadness, and anger; (2) associations between these profiles and demographic variables (i.e., child’s gender, mother’s education, marital satisfaction, and family income); and (3) links between profile membership and adolescent adjustment (emotion regulation and depressive symptoms). Given the paucity of research on discrete emotion socialization and parents’ joint contributions, we took an exploratory stance and formulated the following hypotheses:

**Hypothesis** **1:** *Profile enumeration. At least four parental emotion-socialization profiles would emerge: three consistent (supportive, unsupportive, disengaged) and one discrepant pattern in which mothers and fathers adopt divergent strategies (i.e., a supportive father with an unsupportive mother, or a disengaged father with an engaged mother). Further, the consistent profiles would emerge across all emotional conditions, whereas the discrepant profile would be most pronounced during happiness socialization.*

**Hypothesis** **2:** *Demographic correlates. Parents of daughters (especially in sadness contexts), as well as those with higher income and education, would be more likely to belong to the Consistent Supportive profile.*

**Hypothesis** **3:** *Adjustment differences. Compared with other profiles, adolescents from families with consistently supportive parents would demonstrate superior emotion regulation and the lowest levels of depressive symptoms.*

## 2. Materials and Methods

### 2.1. Participants

This study included 666 adolescents from a middle school in Guangdong Province, China, and their mothers (*n* = 513). The discrepancy between the number of adolescents and mothers in our study is primarily attributed to some mothers not returning their questionnaires promptly due to various reasons, such as time constraints or lower educational levels. Adolescents consisted of 7th-grade students, including 362 boys and 304 girls, with an average age of 13.16 years. The monthly income of most families (54.69%) was CNY 6000 or less, and nearly half (49.80%) of mothers had an education level of junior high school or below. Details for demographic information can be seen in Table 1.

### 2.2. Procedures

This study collaborated with a middle school where teachers introduced the research and invited students and their fathers and mothers to participate. Parents signed the Parent/Guardian and Adolescent Informed Consent Form if they agreed to both their own and their adolescent’s participation in the survey. After obtaining approval from the parents and schools, trained research assistants distributed the informed assent and monitored the completion of adolescent questionnaires (i.e., paternal and maternal response to discrete emotion, emotion regulation, and depressive symptoms) in classrooms, which took approximately 30 min. Adolescents then took home questionnaires for their parents to complete; to maximize sample size and data completeness, we used demographic data reported by mothers. The protocol was approved by the Shaoguan University Ethics Committee, and participants received a small gift upon returning fully completed surveys.

### 2.3. Measurements

#### 2.3.1. Parental Reactions to Adolescents’ Happiness, Sadness, and Anger

We used the 90-item Emotions as a Child Negative and Positive Scales, Version 2 (EAC2; Garside, 2004), and the happiness, sadness, and anger subscales were included (30 items each). Adolescents rated maternal and paternal responses to their emotions on a 1 (never) to 5 (always) scale. Except for interchanging “mother” with “father,” the wording of the items did not vary. After obtaining email authorization from the original developers, the English version of the EAC2 was translated using the back-translation technique.

The original version of the scale comprised five dimensions: reward (comfort or encouragement), magnify (intensifying expressions), punish (derogation), override (providing distraction to redirect attention), and neglect (ignoring). Exploratory factor analysis (EFA) showed a three-factor model for each subscale, and items and the structure were shown in Table 2. One item (“not like my being sad”) was dropped due to a low loading (<0.30). Cronbach’s alpha showed good reliability for all factors except sadness magnify (0.54), likely due to its two-item composition. This factor was retained for analysis. Guided by Gottman’s conceptualization (Gottman et al., 1996), the factors of the sadness subscale were labeled “coaching (comfort and soothe),” “dismissing (ignoring and derogating),” and “magnifying (being distressed).” Similarly, the anger subscale factors were labeled “coaching,” “dismissing,” and “neglect.” For the happiness subscale, the factors were labeled “enhancing (acceptance),” “dampening (suppressing),” and “neglect (ignoring)” following prior studies (Shi et al., 2024b). 

#### 2.3.2. Marital Satisfaction

Mothers rated their marital satisfaction on a 0–70 visual analog scale (0 = extremely unsatisfied; 70 = extremely satisfied; Locke & Wallace, 1959).

#### 2.3.3. Emotion Regulation

Adolescents reported their emotion regulation ability using the Children’s Emotion Management Scale: Sadness and Anger Scales (Zeman et al., 2001). Each subscale comprises three dimensions (i.e., coping, inhibition, and dysregulation), and items are rated on a scale from 1 (hardly ever) to 3 (often). In the current study, the emotion coping dimension was included, with five items for sadness (i.e., “I stay calm and don’t let sad things get to me”) and four items for anger (i.e., “When I’m angry, I stay calm and keep my cool”). Internal consistency was satisfactory (Cronbach’s α = 0.83 for sadness; α = 0.86 for anger), and the two coping scores (*r* = 0.67) were averaged into a composite regulation score, with higher values indicating better regulation.

#### 2.3.4. Depressive Symptoms

Adolescents’ depressive symptoms were measured by the 13-item DSM-Oriented Affective Problems Scale from the Youth Self-Report (Achenbach et al., 2001). Adolescents rated their symptoms on a 3-point scale ranging from 0 (“not true”) to 2 (“very true”). Higher scores indicated greater depressive symptoms (Cronbach’s α = 0.89). 

### 2.4. Data Analysis

The data was analyzed via SPSS 27.0, Rstudio 4.5, and Mplus 8.11. In SPSS 27.0, we computed descriptive statistics (means, standard deviations, Pearson’s correlations, and paired-sample *t*-tests) for the focal variables and conducted an EFA of the EAC2. Visualizations such as a heatmap and forest plot were produced with RStudio 4.5.

All subsequent mixture models were carried out in Mplus 8.11. Step 1. LPA was conducted to identify profiles of parental reactions to adolescents’ happiness, sadness, and anger. The fit indices included the Akaike Information Criterion (AIC), Bayesian Information Criterion (BIC), sample size-adjusted BIC (aBIC), Bootstrapping Likelihood Ratio Test (BLRT), Vuong–Lo–Mendell–Rubin Likelihood Ratio Test (VLMR), and entropy. The following criteria determined the superior model: (a) lower AIC, BIC, and aBIC, (b) significant BLRT and VLMR, and (c) higher entropy (0.80). Step 2. Logistic regression was performed using the AUXILIARY command (R3STEP) to model demographics (gender, education, income, and marital satisfaction) as predictors of profile membership (Asparouhov & Muthén, 2014). Step 3. Differences in the outcome across profiles were examined with the manual Bolck–Croon–Hagenaars approach, adjusting for covariates by (a) saving the profile membership probabilities, and (b) testing the regression models with the TRAINING and MODEL TEST commands (McLarnon & O’Neill, 2018). 

## 3. Results

### 3.1. Descriptive Statistics

**Figure 1:**
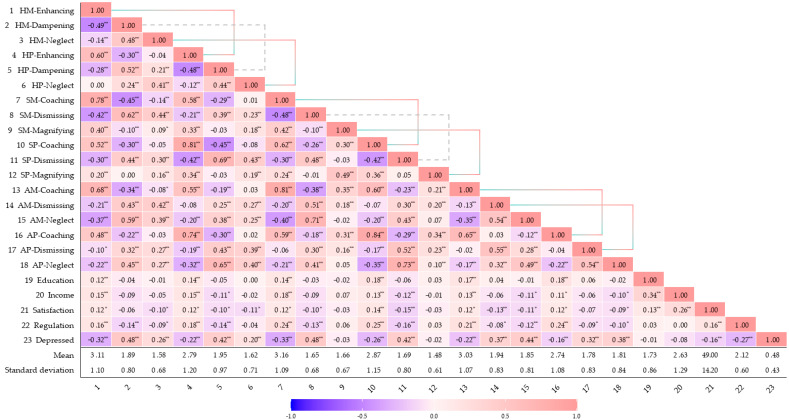
Correlation heatmap and mother–father emotion socialization differences. Note: connecting lines represent differences in ES between fathers and mothers as perceived by adolescents (gray lines denote marginal significance); H = happiness; S = sadness; A = anger; M- = maternal; P- = paternal. ** p < 0.01. * p < 0.05.

Pearson’s correlations showed that enhancing (happiness) and coaching (anger, sadness) relate to better emotion regulation, fewer depressive symptoms, and higher SES, whereas dampening, neglect, and dismissing show the opposite (see Figure 1). Besides, the gender difference showed mothers displayed more enhancing (happiness), coaching (sadness and anger), magnifying (sadness), and dismissing (anger) responses, but less neglect (happiness) compared with fathers.

### 3.2. Latent Profile Analysis of Parental Reactions to Emotions 

#### 3.2.1. Profiles of Parental Reactions to Happiness

**Figure 2:**
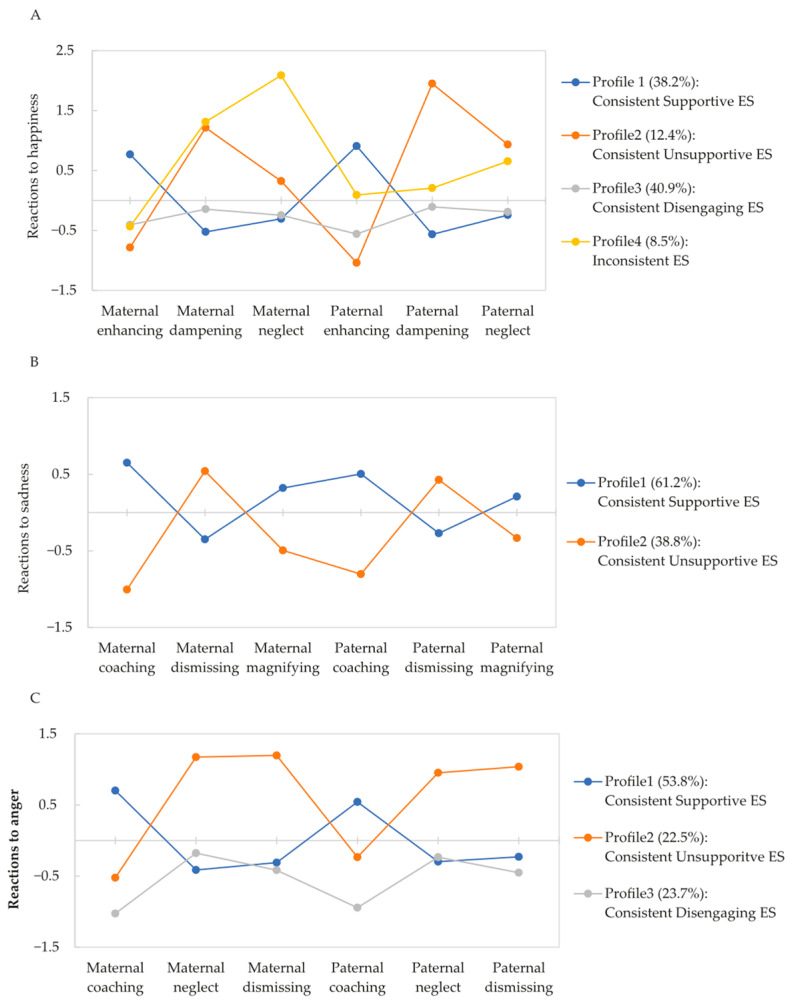
Standardized means of parental emotion socialization varied by profile. (**A**) Profiles of paternal and maternal reactions to adolescents’ happiness. (**B**) Profiles of paternal and maternal reactions to adolescents’ sadness. (**C**) Profiles of paternal and maternal reactions to adolescents’ anger.

The model fit results for parental reactions to happiness are shown in Table 3. The AIC, BIC, and aBIC values all decreased as the number of profiles increased, whereas the VLMR p-value indicated that the three- and five-profile models failed to improve the model fit. Both the two- and four-profile models produced entropy values above 0.80. However, the AIC, BIC, and aBIC values were lower in the four-profile model than in the two-profile model. Therefore, a four-profile model was selected. As shown in Figure 2A, three profiles displayed consistent reactions between fathers and mothers, indicating that adolescents generally perceive both parents as using similar ES strategies. Specifically, the first profile featured high enhancing but low dampening and neglect, which was labeled as Consistent Supportive ES (38.2%). Characterized by low enhancing, as well as above-average dampening and neglect, the second profile was labeled Consistent Unsupportive ES (12.4%). The third profile was labeled Consistent Disengaging ES (40.9%), with the means of all indicators below zero. In the fourth profile, maternal and paternal ES behaviors differed remarkably and were labeled as Inconsistent ES (8.5%). Mothers were perceived as having high levels of dampening and neglect, whereas fathers were perceived as having low levels on average for all indicators for adolescents. 

#### 3.2.2. Profiles of Parental Reactions to Sadness and Anger

The two-profile model was selected as the best-fit model for parental reactions to sadness (Table 3). As shown in Figure 2B, most families were classified in the Consistent Supportive ES profile (61.2%); both parents in the profile exhibited high coaching and magnifying but low dismissing. The rest were classified as the Consistent Unsupportive ES profile (38.8%); both parents showed high coaching and magnifying but low dismissing. Similarly, regarding the parental reactions to anger, the three-profile model was selected based on the aforementioned criteria (Table 3). As expected, the first two profiles were labeled Consistent Supportive ES (53.8%) and Consistent Unsupportive ES (22.5%). The third profile, characterized by low levels of all indicators, was labeled as Consistent Disengaging ES (53.8%; Figure 2C). 

### 3.3. Child Gender, Mothers’ Education, Marital Satisfaction, and Income Correlates 

A series of logistic regressions were conducted using the R3STEP, with the Consistent Supportive ES profile set as the reference group, as it was the most common pattern identified across LPA models (Table 4). For the happiness condition, the results showed that low levels of family income were associated with greater odds of parents being classified in the Consistent Disengagement profile than in the Consistent Supportive profile (*OR* = 0.79, *p* < 0.05). Similarly, low levels of marital satisfaction were associated with greater odds of parents being classified in the Consistent Unsupportive ES profile (*OR* = 0.98, *p* < 0.05). For the sadness condition, the results showed that girls were more likely than boys to perceive their parents as fitting the Consistent Unsupportive ES profile rather than the Consistent Supportive ES profile (*OR* = 1.88, *p* < 0.05). For the anger condition, the results showed that girls (*OR* = 2.22, *p* < 0.05) and low levels of mothers’ education (*OR* = 0.60, *p* < 0.05) were associated with greater odds of parents being classified in the Consistent Disengaging ES profile than in the Consistent Supportive ES profile. Comparisons between the Consistent Supportive ES profile and the other profiles did not significantly differ in terms of adolescents’ gender, mothers’ education, marital satisfaction, and family income.

### 3.4. Latent Profiles and Depressive Symptoms and Emotion Regulation

As shown in Table 5, we compared the differences in adolescents’ emotional adjustment after adjusting for demographics. When adolescents perceived their parents to be in the Consistent Supportive profile, they showed optimal emotional adjustment (i.e., lowest levels of depressive symptoms and highest levels of emotion regulation) for all emotional conditions. In contrast, adolescents who perceived their parents to be in the Consistent Unsupportive profile demonstrated the poorest emotional functioning, especially in response to sadness. Findings for the Consistent Disengaging profile varied by emotion. In the happiness condition, these adolescents reported lower depressive symptoms and better emotion regulation than those perceiving a Consistent Supportive profile; under the anger context, however, depressive symptom levels did not differ between the two groups, suggesting that parental disengagement in response to anger may be less detrimental. Finally, adolescents perceiving an Inconsistent profile experienced higher depressive symptoms than those perceiving a Consistent Disengaging profile, while their emotion regulation and overall symptom levels were similar to those of the Consistent Unsupportive profile.

## 4. Discussion

The current study used LPA to identify profiles of how Chinese parents jointly react to adolescents’ discrete emotions (happiness, sadness, and anger), and examined familial, parent, and child correlates of these profiles. Results generally supported our initial hypothesis: most families exhibited similar maternal and paternal reactions within each emotional context (i.e., both supportive, both unsupportive, or both disengaging), while a subset exhibited divergent parental responses. Interestingly, reaction patterns varied by emotion: both parents tended to disengage in response to happiness and anger, but not sadness. In addition, the divergent reaction pattern, characterized by unsupportive mothers and disengaging fathers, emerged exclusively for happiness. These patterns were associated with demographic factors and linked to adolescents’ emotional adjustment at varying levels, underscoring the need for tailored, emotion-specific interventions that account for family context to optimally support adolescent well-being.

### 4.1. Profile Enumeration of Socialization of Discrete Emotions

Consistent with Hypothesis 1, we captured parental consistency of ES across all emotional conditions, including the Consistent Supportive and Consistent Unsupportive profiles, which reflects the similar reactions between fathers and mothers regardless of the emotion types. These findings align with previous studies on parental reactions to negative emotions (Miller-Slough et al., 2018; Zhu & Dunsmore, 2023), and expand our understanding of how adolescents’ happiness is socialized. The general similarity between fathers and mothers may be explained by assortative mating theory, which suggests that partners with similar attitudes and beliefs tend to select each other (Lansford et al., 2014). Once in a relationship, partners also tend to influence each other and become more similar over time. Therefore, as parents operate as a coparenting unit, they may adopt each other’s parenting practices and react consistently to their children’s emotional expressions, for better or worse. Additionally, mothers and fathers may show similarities in parenting because they are responding to the stimulus of the same child. Finally, shared method variance due to adolescents reporting on both mothers’ and fathers’ behaviors might have contributed to cross-parent similarities on our measures (Jager et al., 2016).

However, this consistency did not extend to parents’ disengaging responses, which emerged only for happiness and anger but not for sadness, labeled as the Consistent Disengaging profile (relatively low across measures). This pattern aligns with Russell’s circumplex model, as both happiness (high pleasure, high arousal) and anger (low pleasure, high arousal) share heightened arousal and potential for interpersonal contagion. In collectivist cultures such as China, where interpersonal harmony is strongly emphasized, high-arousal emotions are often viewed as disruptive or inconsiderate of others’ feelings, potentially provoking jealousy or gossip (Aggarwal et al., 2022; Malatesta & Wilson, 1988; O’Neal & Magai, 2005). As a result, emotionally reserved Chinese parents may adopt a passive approach to managing their children’s happiness and anger, not merely relying on direct suppression, reflecting a broader range of subtle emotional management strategies aimed at maintaining cohesion. In contrast, sadness is a nonhostile emotion with low arousal that tends to elicit engagement (i.e., either supportive or non-supportive) rather than withdrawal (Barett & Campos, 1987), which may explain the absence of a disengaging profile in the sadness condition. Future studies should examine whether cultural factors, such as collectivist orientation or sensitivity to emotional contagion, contribute to parents’ passive disengagement from high-arousal emotions. The implications of such disengagement may differ between happiness and anger, which will be explored in the following subsection.

Beyond these general similarities, we also expected and identified an Inconsistent ES profile marked by divergent maternal and paternal reactions exclusively in the happiness condition (not in the sadness or anger). This finding aligns with Lunkenheimer et al. (2012), whose laboratory observations revealed that parents exhibit greater flexibility and employ a wider range of strategies when responding to children’s expressions of happiness compared to negative feelings. From a functionalist perspective, happiness represents a desirable state that triggers “free activation,” encouraging spontaneous and unplanned interactions (Aggarwal et al., 2022; Fredrickson, 2013). In contrast, sadness and anger are often treated as “problem emotions” that require parents’ special attention or additional intervention and call for more uniform and goal-directed responses to their children’s distress (Forgas, 2017; Majdandžić et al., 2012). Consistent with this interpretation, Crockenberg and Leerke’s (2003) suggest that distressed children may elicit intensified parental cooperation, prompting parents to align their approaches to downregulate negative emotions. Therefore, the unique divergence observed in responses to happiness likely reflects the lower perceived necessity for coordinated interventions and highlights the spontaneous and individualized nature of parental reactions in positive emotional contexts.

Furthermore, this divergence profile (characterized by low scores on all paternal reactions and high maternal dampening and neglect reactions) reflects paternal disengagement and maternal nonsupport, which was unexpected and has not been fully captured by those studies on parents’ collaborative efforts. Nevertheless, researchers focusing on either fathers or mothers individually suggest that paternal disengagement (from negative emotions) and maternal nonsupport (toward positive emotions) are relatively common in Chinese families (Shi et al., 2024b; Song et al., 2019; Wang et al., 2019). These findings may be better understood from a cultural lens. In collectivist contexts, emotional restraint is valued to maintain interpersonal harmony, a tendency especially notable among Chinese fathers, who typically show lower emotional and verbal expressiveness than mothers (Li, 2021). Given that positive emotions like happiness generally do not prompt parental intervention in the same manner as negative emotions, Chinese fathers might be more inclined toward passive disengagement when responding to happiness. This paternal disengagement can unintentionally increase maternal parenting burdens, often described as “widow-parenting,” a phenomenon reflecting the limitations of traditional gendered divisions of labor (“men in charge of the outside, women in charge of the inside”). Indeed, from the broader parenting literature, prior research has shown that paternal disengagement exacerbates maternal parenting stress (Nomaguchi et al., 2017). Under the heightened parenting responsibilities and difficulties, mothers were less likely to employ supportive strategies to address household chaos and assist children in managing emotions (Cui et al., 2021). This culturally shaped, gender-based dynamic has recently sparked significant debate on Chinese social media (Shen & Jiao, 2024), underscoring the necessity of further empirical investigations to clarify its broader implications for the nuanced interplay among familial roles, cultural values, and adolescent emotion socialization.

### 4.2. Relationships Between ES Patterns and Their Predictors and Outcomes

Logistic regression results indicated that parents with higher income, better education, and greater marital satisfaction were more likely to be in the Consistent Supportive profile and provide more positive reactions to their children, which is consistent with Hypothesis 2 and previous person-centered research (King et al., 2023; Sosa-Hernandez et al., 2020). These results provide further support for spillover theory, which posits that positive marital interactions and advantageous socioeconomic circumstances extend into the parent–child relationship, enhancing parents’ emotional resources and facilitating more adaptive parental responses toward their children. Although parents’ greater support for boys’ anger replicates Radke-Yarrow and Kochanska’s (1990) observation that boys’ expressions of anger are met with relative tolerance, our finding that boys also received more support than girls when displaying sadness requires a broader interpretation of gender norms, developmental vulnerability, and cultural change. In many families, a boy’s sadness may violate prevailing expectations of male stoicism, rendering his distress unusually salient and prompting parents to respond with heightened empathy and practical assistance (Fabes & Martin, 1991). During adolescence in particular, parents may perceive boys as less practiced at articulating or regulating sadness and therefore intervene more readily to alleviate perceived vulnerability (Raval & Walker, 2019). At the same time, contemporary shifts in Chinese parenting ideals—spurred by urbanization and increased emphasis on emotional competence—have encouraged caregivers to support boys’ emotional expression, including sadness, as part of healthy socioemotional development (Li, 2021). Therefore, children’s gender may prove an intriguing focus for future longitudinal research to explore these dynamics further.

Generally speaking, adolescents with consistently supportive ES showed the best emotional adjustment, whereas those with parents with consistently unsupportive ES showed the poorest, supporting our Hypothesis 3 and prior work (Miller-Slough et al., 2018; Yu et al., 2015; Zhu & Dunsmore, 2023). Furthermore, we found parents in the Inconsistent profile were associated with greater depressive symptoms and emotion dysregulation, aligning with the results of Yu et al. (2015), who used the variable-centered approach. This might be because inconsistent reactions of parents convey mixed messages that undermine emotional security, particularly during adolescence, a critical period of emotional instability and self-identity development. Unexpectedly, adolescents whose parents employed disengaging strategies (Consistent Disengaging profile) exhibited more depressive symptoms in the happiness condition, whereas the opposite was true for anger. Functionalists argue that happiness is typically shared to gain acceptance (Barett & Campos, 1987). Thus, disengaging or unsupportive reactions might greatly suppress adolescents’ desire to communicate these feelings, increasing the risk for dysregulation and depression (Katz et al., 2014; Yap et al., 2008; Yi et al., 2016). In addition, adolescents’ egocentrism heightens their sensitivity to others’ appraisals, increasing the likelihood of perceiving disengagement as rejection (Rohner & Lansford, 2017). Such cognitive tendencies potentially shape interactions. In contrast, parental disengagement from adolescents’ anger may have a constructive role (Vanwoerden et al., 2022), especially in collectivist contexts that value interpersonal harmony. Specifically, anger, characterized by high arousal and unpleasantness, can trigger interpersonal contagion and escalate conflicts if not properly managed (Russell, 1980). An interview study of Asian mothers found that those with healthy children (rather than those with maladjusted children) were more likely to use a “not talking to the child for a brief period” strategy when their children expressed anger. They perceived it as beneficial because this approach provides adolescents with some time to reflect and allows parents to calm down and think about what to do (Raval & Martini, 2011). Our findings echo this culturally framed approach, indicating that parental disengagement during anger may promote adolescent self-regulation and relational repair. Future studies should examine how such culturally informed disengagement tactics influence long-term emotion regulation and family dynamics.

### 4.3. Limitations and Implications

This study has several limitations. First, the sole reliance on adolescent self-reports of ES may inflate shared method variance and obscure genuine differences between maternal and paternal behaviors; future studies should triangulate adolescents’ perceptions with father- and mother-reports or employ observational and ecological-momentary assessment methods to reduce informant bias. Second, we assessed marital satisfaction only from mothers’ perspectives, thereby offering an incomplete portrait of the couple’s relational climate; incorporating paternal self-reports and behavioral coding of partner interactions would enhance the validity of family-level analyses. Third, the cross-sectional design precludes any inference regarding temporal precedence or causality between emotion-socialization strategies and adolescent adjustment; longitudinal or intervention-based research is necessary to establish directional effects and developmental trajectories. Finally, our sample was drawn from a single middle school in Guangdong Province, which may constrain the generalizability of results; replication with more diverse samples across different cultural, socioeconomic, and geographic settings is therefore recommended.

Our identification of distinct parental emotion profiles yields two primary implications for practice. The first concerns joint, emotion-specific parent training: programs should involve both mothers and fathers and tailor content to discrete emotions. For sadness and anger, modules can teach soothing techniques and empathic listening to alleviate distress; for happiness, activities should guide parents toward a shared response strategy, fostering consensus and reinforcing positive interactions. The second involves strategic disengagement for anger management: when addressing anger, parents and adolescents benefit from techniques such as “not for talking for a brief period”—brief pauses in communication that give adolescents space to self-regulate before conflicts escalate. Counseling centers can incorporate these methods into parent-education curricula, training caregivers to recognize and implement temporary disengagement as a constructive emotion regulation tool.

## Figures and Tables

**Table 1:** Demographic Information in the Current Study.

	Frequency	%
Gender		
boys	362	54.35
girls	304	45.65
Education		
≤middle school	255	38.29
senior secondary school or high school	162	24.32
community college	74	11.11
≥bachelor	21	3.15
Monthly Income		
≤2999	98	14.71
3000–5999	176	26.43
6000–8999	101	15.17
9000–14,999	85	12.76
15,000–19,999	23	3.45
≥20,000	18	2.70

**Table 2:** The factorial structure and items of EAC2 used in the current study.

Happiness					Sadness					Anger				
ORIG. Structure	Items	Factors			ORIG. Structure	Items	Factors			ORIG. Structure	Items	Factors		
		h1	h2	h3			s1	s2	s3			a1	a2	a3
REWARD	interested in why I was happy				REWARD	asked me about it				REWARD	found out what made me angry			
	encouraged me to share					deal with the problem					deal with the problem			
	listened to me					comforted me					understood why I was angry			
MAGNIFY	said he/she was happy, too				OVERRIDE	told me not to worry				OVERRIDE	told me things were not so bad			
	became very happy/themselves					told me to cheer up					told me to change my attitude			
	joined me in my happiness					bought something I like					told me to keep quiet			
PUNISH	not like my being happy				PUNISH	not like my being sad ^a^					said I should be ashamed			
	told me to keep it to myself					gave me a disgusted look				PUNISH	punished me			
	told me to settle down					called me a crybaby					gave me something else to do			
OVERRIDE	other things were more important				NEGLECT	usually was not around				NEGLECT	ignored			
	changed the subject					most times did not notice					most times did not notice			
	quickly moved on to something else					ignored me					usually was not around			
NEGLECT	usually did not notice				MAGNIFY	became sad themselves				MAGNIFY	yelled back at me			
	usually was not around					became upset					became angry at me			
	failed to ask about it					became tearful or cried					became upset at me			

Note. Gray shading indicates factor loadings ≥0.40; ORIG. = the original version of the five-factor scale; h1 = enhancing (α_m_ = 0.90, α_f_ = 0.92); h2 = dampening (α_m_ = 0.67, α_f_ = 0.65); h3 = neglect (α_m_ = 0.80, α_f_ = 0.84); s1 = coaching (α_m_ = 0.90, α_f_ = 0.91); s2 = dismissing (α_m_ = 0.69, α_f_ = 0.76); s3 = magnifying (α_m_ = 0.54, α_f_ = 0.54); a1 = coaching (α_m_ = 0.86, α_f_ = 0.86); a2 = neglect (α_m_ = 0.72, α_f_ = 0.71); a3 = dismissing (α_m_ = 0.75, α_f_ = 0.78). ^a^ The item was eliminated and not included in the subsequent analysis.

**Table 3:** LPAs of parental socialization of different emotions.

No. Profiles	AIC	BIC	aBIC	Entropy	VLMR *p*-Value	BLRT *p*-Value
**ES in the Happiness**						
1 Profile	11,071.855	11,125.799	11,087.698	–	–	–
2 Profiles	10,410.665	10,496.075	10,435.750	0.866	0.012	<0.001
3 Profiles	10,170.740	10,287.617	10,205.066	0.756	0.347	<0.001
**4 Profiles**	**9969.349**	**10,117.692**	**10,012.916**	**0.803**	**0.035**	**<0.001**
5 Profiles	9840.625	10,020.436	9893.434	0.806	0.168	<0.001
**ES in the Sadness**						
1 Profile	11,054.828	11,108.771	11,070.671	–	–	–
**2 Profiles**	**10,387.570**	**10,472.980**	**10,412.655**	**0.818**	**<0.001**	**<0.001**
3 Profiles	10,143.238	10,260.115	10,177.564	0.835	0.132	<0.001
4 Profiles	9912.591	10,060.935	9956.159	0.847	0.469	<0.001
5 Profiles	9795.686	9975.497	9848.495	0.839	0.243	<0.001
**ES in the Anger**						
1 Profile	11,066.180	11,120.123	11,082.022	–	–	–
2 Profiles	10,434.814	10,520.224	10,459.899	0.831	<0.001	<0.001
**3 Profiles**	**10,135.329**	**10,252.206**	**10,169.655**	**0.806**	**0.009**	**<0.001**
4 Profiles	9938.495	10,086.839	9982.063	0.824	0.094	0.939
5 Profiles	9809.767	9989.577	9862.576	0.838	0.503	<0.001

Note. Optimal models for each emotion type are highlighted in bold. AIC = Akaike information criterion; BIC = Bayesian information criterion; aBIC = adjusted BIC; LMR = Lo-Mendell-Rubin adjusted likelihood ratio test; BLRT = Bootstrapped likelihood ratio test.

**Table 4:** Logistic regressions across emotion types with the 3-step method.

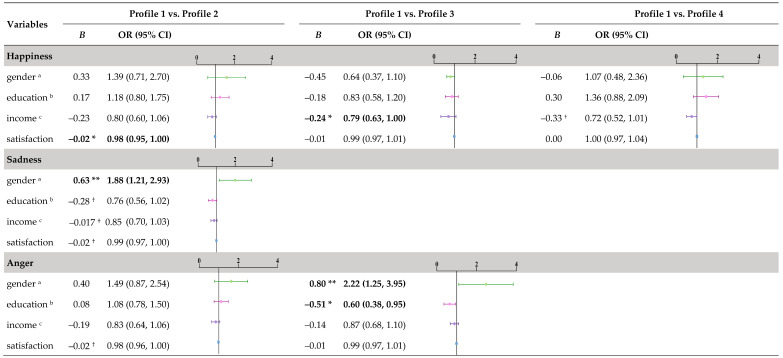

Note. Statistically significant differences are denoted in bold. The consistent supportive ES was the reference group; Profile 1 = consistent supportive ES; Profile 2 = consistent unsupportive ES; Profile 3 = consistent disengagement ES; Profile 4 = inconsistent ES; satisfaction = marital satisfaction. ^a^ 0 = male, 1 = female. ^b^ 1 = middle school or below, 2 = senior secondary school or high school, 3 = community college, 4 = bachelor or above; ^c^ 1 = ≤2999, 2 = 3000–5999, 3 = 6000–8999, 4 = 9000–14,999, 5 = 15,000–19,999, 6 = ≥20,000. ^†^ *p* < 0.10. * *p* < 0.05. ** *p* < 0.01.

**Table 5:** Differences in the outcomes across the profiles of ES with the manual BCH method.

Outcomes	Happiness				Sadness				Anger			
	Profile	*M*	χ^2^(3)	Diff	Profile	M	χ^2^(1)	Diff	Profile	*M*	χ^2^(2)	Diff
Emotion regulation			13.66 **	P1 > P3 = P4 = P2			20.98 ***	P1 > P2			21.09 ***	P1 > P2 = P3
	P1	2.09			P1	2.06			P1	2.09		
	P2	1.89			P2	1.79			P2	1.84		
	P3	1.85							P3	1.80		
	P4	1.91										
Depressive symptoms			59.41 ***	P1 < P3 < P4 = P2			27.80 ***	P1 < P2			65.80 ***	P1 = P3 < P2
	P1	0.39			P1	0.45			P1	0.43		
	P2	0.84			P2	0.71			P2	0.91		
	P3	0.55							P3	0.48		
	P4	0.81										

Note. Covariates include gender, education, income, and marital satisfaction; P1 = consistent supportive ES; P2 = consistent unsupportive ES; P3 = consistent disengagement ES; P4 = inconsistent ES. ** *p* < 0.01. *** *p* < 0.001.

## Data Availability

The data presented in this study are available on request from the corresponding author.
